# Inhibition of insect olfactory behavior by an airborne antagonist of the insect odorant receptor co-receptor subunit

**DOI:** 10.1371/journal.pone.0177454

**Published:** 2017-05-31

**Authors:** Devin Kepchia, Scott Moliver, Kunal Chohan, Cameron Phillips, Charles W. Luetje

**Affiliations:** Department of Molecular and Cellular Pharmacology, University of Miami Miller School of Medicine, Miami, Florida, United States of America; Plant and Food Research, NEW ZEALAND

## Abstract

Response to volatile environmental chemosensory cues is essential for insect survival. The odorant receptor (OR) family is an important class of receptors that detects volatile molecules; guiding insects towards food, mates, and oviposition sites. ORs are odorant-gated ion channels, consisting of a variable odorant specificity subunit and a conserved odorant receptor co-receptor (Orco) subunit, in an unknown stoichiometry. The Orco subunit possesses an allosteric site to which modulators can bind and noncompetitively inhibit odorant activation of ORs. In this study, we characterized several halogen-substituted versions of a phenylthiophenecarboxamide Orco antagonist structure. Orco antagonist activity was assessed on ORs from *Drosophila melanogaster* flies and *Culex quinquefasciatus* mosquitoes, expressed in *Xenopus laevis* oocytes and assayed by two-electrode voltage clamp electrophysiology. One compound, OX1w, was also shown to inhibit odorant activation of a panel of *Anopheles gambiae* mosquito ORs activated by diverse odorants. Next, we asked whether Orco antagonist OX1w could affect insect olfactory behavior. A *Drosophila melanogaster* larval chemotaxis assay was utilized to address this question. Larvae were robustly attracted to highly diluted ethyl acetate in a closed experimental chamber. Attraction to ethyl acetate was Orco dependent and also required the odorant specificity subunit Or42b. The addition of the airborne Orco antagonist OX1w to the experimental chamber abolished larval chemotaxis towards ethyl acetate. The Orco antagonist was not a general inhibitor of sensory behavior, as behavioral repulsion from a light source was unaffected. This is the first demonstration that an airborne Orco antagonist can alter olfactory behavior in an insect. These results suggest a new approach to insect control and emphasize the need to develop more potent Orco antagonists.

## Introduction

Olfaction, the sensing of airborne chemicals from the environment, is a critical process for insects, allowing detection of food, danger and mates. Importantly, olfaction allows disease vector insects to locate and feed on humans [1–3]. Odorant molecules are detected by members of several chemosensory receptor families, including the olfactory receptors (ORs) that are embedded in the plasma membranes of olfactory sensory neurons (OSNs) located in the antennae and maxillary palps [2]. Insect ORs are ligand (odorant) gated nonselective cation channels [4, 5]. These receptors have also been proposed to initiate, or be modified by, second messenger cascades [5, 6]. Insect ORs are heteromeric complexes composed of a variable odorant specificity subunit and a constant odorant receptor co-receptor (Orco) subunit, in an unknown stoichiometry [7–9]. Both the odorant specificity and Orco subunits contribute to the properties of the channel pore [10–12], while the odorant specificity subunits are the major determinant of odorant sensitivity [13–18]. Numerous odorant specificity subunits are expressed within a species: for example, 62 in *D*. *melanogaster* [8], 79 in *A*. *gambiae* [18], and 176 in *C*. *quinquefasciatus* [19]. In contrast, each species expresses a single, highly conserved Orco subunit [9, 20–24]. Some ORs are highly specialized, focusing on specific molecules such as pheromones [25] or various ecologically relevant odorants [26, 27]. Other ORs appear to be part of a combinatorial coding system in which each odorant activates multiple ORs and each OR is activated by multiple odorants [13, 14, 18]. Extensive divergence of the odorant specificity subunit family allows each species to survey ecologically relevant portions of odor space to guide behavioral decisions [13].

A major approach to controlling the spread of insect-borne disease is the use of insect repellents. *N*,*N*-diethyl-*m*-toluamide (DEET) is the active ingredient in most current insect repellents, acting as an airborne spatial repellent and as a contact irritant [28, 29]. Additional repellents have been developed, such as p-menthane-3,8-diol (PMD), IR3535 and picaridin, but none are as effective as DEET [29]. However, DEET is not without its drawbacks. It must be used at high concentrations, ranging from 7% (350 mM) to as much as 98% (5 M), and requires frequent reapplication. This makes DEET too expensive for daily use in regions most affected by insect-borne disease [29]. Additionally, mosquitoes can exhibit decreased repellency by DEET following previous exposure [30]. While contact effects of DEET are mediated by gustatory receptors [31, 32], the spatial repellency is OR dependent [33], making the ORs attractive targets for the development of new repellents.

Most recent efforts to target ORs for the development of new repellent compounds have involved identification of odorant specificity subunits that recognize behaviorally important odorants [13, 18, 34–37], and in a few cases, subsequent large-scale ligand screening [38, 39]. However, odorant specificity subunit families are quite divergent across species and there is variation in the odorants and odorant specificity subunits that are important for various species-specific behaviors [2, 40]. In addition, OSN responsiveness and olfactory behavior in mosquitoes is altered after a blood meal or following infection by the malaria parasite [41–43]. Changes in the levels of antennal RNA transcripts encoding odorant specificity subunits have been observed after blood feeding in *A*. *gambiae* [44], which may underlie blood meal induced physiological and behavioral changes. This makes the odorant specificity subunits a complex and highly variable set of targets for the development of new insect control agents.

In contrast, each species expresses a single Orco subunit that is present in all ORs and is highly conserved across species [20, 21, 23, 24, 45]. Genetic deletion or suppression of Orco abolishes OR-mediated behaviors in various insects [21, 46, 47] and decreases preference for humans in *Aedes aegypti* mosquitoes [33]. The discovery of a compound, N-(4-ethylphenyl)-2-((4-et-5-(3-pyridinyl)-4H-1,2,4-triazol-3-yl)thio)acetamide (VUAA1), that activates insect ORs through the Orco subunit, revealed the presence of a ligand-binding site on Orco [38]. It is currently unclear whether this binding site has a physiological purpose, but several additional agonists and numerous antagonists of this site have been identified [48–52]. Interestingly, several trace amines have been shown to be potent antagonists of Orco [50]. In addition to blocking activation of ORs by Orco agonists, Orco antagonists have been shown to inhibit odorant activation of a broad range of ORs through an allosteric mechanism [48–52], suggesting that chemical inhibition of OR-mediated behaviors may be possible by antagonizing the Orco subunit. The targeting of Orco offers the possibility of developing compounds that are active at multiple ORs across different species. Here, we show that an airborne Orco antagonist can abolish an olfactory behavior in *D*. *melanogaster* larvae.

## Results and discussion

### Orco antagonist OX1w inhibits odorant activation of a broad range of ORs

**Fig 1:**
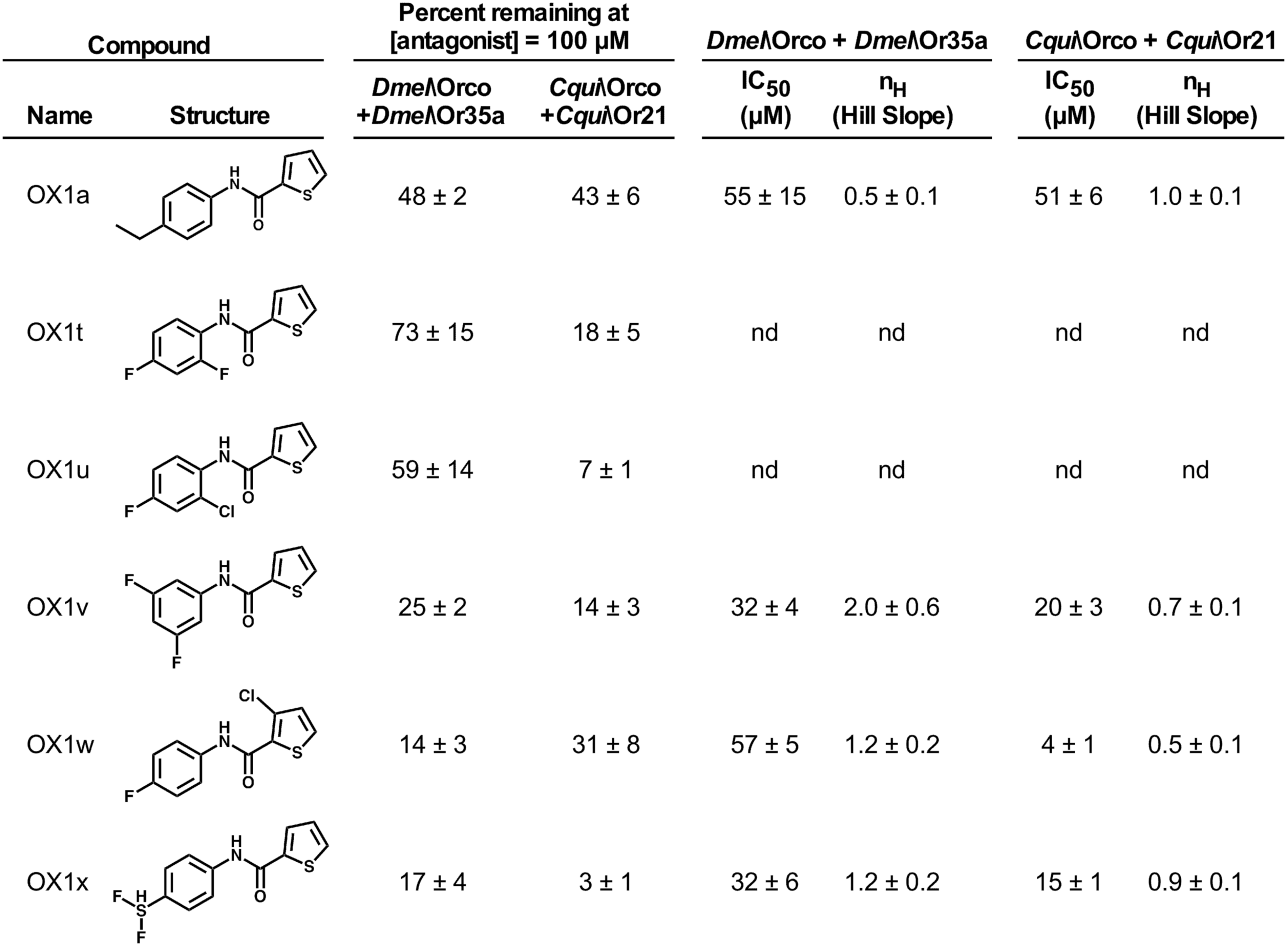
Orco antagonist activity of phenylthiophenecarboxamide compounds OX1t-OX1w. Compounds were initially screened at 100 μM against ORs from *Drosophila melanogaster* (Dmel\Orco+Dmel\OR35a activated by 10 μM OLC12) and *Culex quinquefasciatus* (Cqui\Orco+Cqui\OR21 activated by 3 μM OLC12). Concentration-inhibition curves were constructed for compounds that displayed favorable antagonist activity at both receptors. OX1a (a previously identified Orco antagonist [49]) served as a reference compound. Data are presented as mean ± SEM (n = 3–11). nd, not determined.

The phenylthiophenecarboxamide compound OX1a (Fig 1) is a useful lead structure for the discovery of potent Orco antagonists [48, 49]. We screened a new series of compounds based on the OX1a structure (Fig 1). Various halogen substitutions were made as an attempt to increase potency while maintaining or improving the low volatility of OX1a. We initially screened these compounds, at a concentration of 100 μM, for the ability to antagonize Orco agonist OLC12 [48] activation of ORs (heteromeric complexes of odorant specificity subunits and Orco) expressed in *Xenopus* oocytes and assayed by two-electrode voltage clamp electrophysiology. We chose to use heteromeric ORs as screening targets because this is thought to be the native environment for Orco (as a component of a heteromeric OR complex) and because Orco and odorant specificity subunits exert allosteric effects on one another which can alter the ligand sensitivities of each subunit [48–50]. We used Dmel\Orco+Dmel\Or35a activated by 10 μM OLC12, the approximate EC_25_ [48], as a representative *D*. *melanogaster* OR, because other *D*. *melanogaster* ORs express poorly in *Xenopus* oocytes [11, 16]. As a representative mosquito OR, we used Cqui\Orco+Cqui\Or21 activated by 3 μM OLC12, the approximate EC_25_ [49], from *C*. *quinquefasciatus*, the West Nile Virus vector. OX1v, OX1w, and OX1x showed improved ability to inhibit OLC12 activation of both receptors. A range of concentrations of these three compounds were then tested to allow concentration-inhibition analysis, which revealed OX1w to have substantially improved potency at the mosquito OR, but not at the *D*. *melanogaster* receptor (Fig 1). As our long-term goal is to develop compounds that alter mosquito olfactory behavior, we decided to proceed with OX1w.

**Fig 2:**
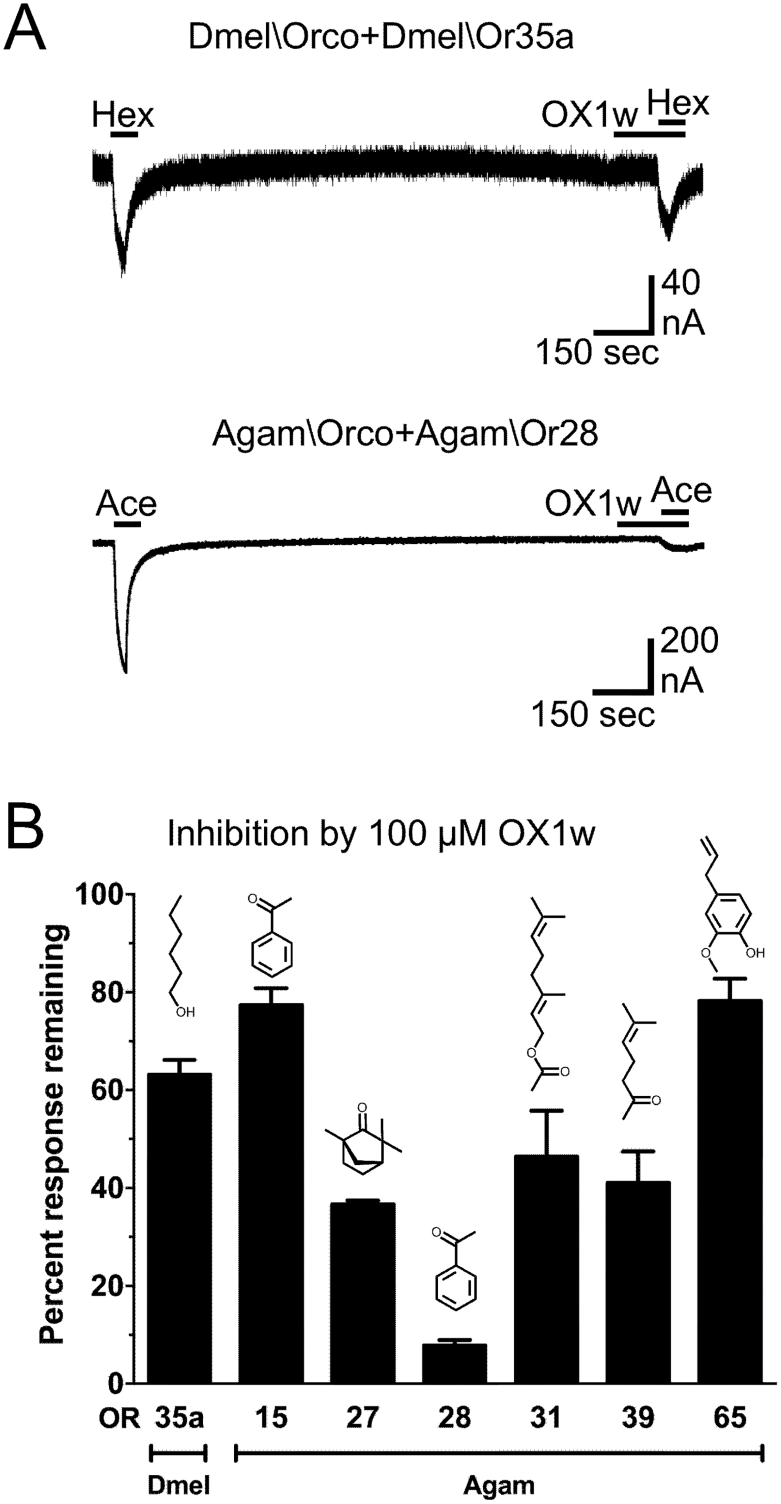
Orco antagonist OX1w inhibits odorant activation of a diverse array of ORs. (A) OX1w inhibits odorant activation of ORs from *D*. *melanogaster* and *A*. *gambiae*. Top trace, an oocyte expressing Dmel\Orco+Dmel\Or35a was challenged with a 30 sec application of 1 μM hexanol (Hex). After a 20 min wash period, 100 μM OX1w was applied for 90 sec before a second application of Hex and coapplied during the Hex application. Bottom trace, an oocyte expressing Agam\Orco+Agam\Or28 was challenged with a 30 sec application of 40 μM acetophenone (Ace). After a 20 min wash period, 100 μM OX1w was applied for 90 sec before a second application of Ace and coapplied during the Ace application. (B) Oocytes expressing a variety of ORs were activated by the appropriate odorant and tested for inhibition by OX1w as in panel A. Dmel\Orco+Dmel\Or35a was activated by 1 μM hexanol, Agam\Orco+Agam\Or15 was activated by 18 μM acetophenone, Agam\Orco+Agam\Or27 was activated by 3 μM L-fenchone, Agam\Orco+Agam\Or28 was activated by 40 μM acetophenone, Agam\Orco+Agam\Or31 was activated by 70 μM geranyl acetate, Agam\Orco+Agam\Or39 was activated by 10 μM 6-methyl-5-hepten-2-one, and Agam\Orco+Agam\Or65 was activated by 100 nM eugenol. Inhibition values were normalized to the value obtained when the assay was run in the absence of OX1w (sham). The structure of each odorant is shown. Data are presented as mean ± SEM (n = 3–8).

Orco antagonists (such as OX1w) are able to block activation by Orco agonists (such as OLC12) when Orco is part of a heteromeric OR (Fig 1) and when Orco forms a homomeric channel (S1 Fig and S6 Table). In addition, several Orco antagonists have also been shown to inhibit odorant activation of ORs through an allosteric mechanism [48–51]. Because Orco is present in all ORs, Orco antagonists could act as universal inhibitors of OR function and might then serve to alter OR-mediated behaviors. To determine whether OX1w could also inhibit odorant activation, we tested 100 μM OX1w against a diverse array of ORs (Fig 2). Dmel\Orco+Dmel\Or35a activation by hexanol (1μM) was partially inhibited by 100 μM OX1w. We also tested OX1w against ORs from *A*. *gambiae*, the malaria vector, because a large set of *A*. *gambiae* ORs displaying robust function in oocytes is available [18]. Six different odorant specificity subunits from *A*. *gambiae* were each co-expressed with Agam\Orco and activated with known odorant ligands for these receptors [18, 50]. Odorants were applied at an EC_50_ concentration to activate each receptor. Repeated odorant application to ORs expressed in *X*. *laevis* oocytes can cause a substantial decrease in the amplitude of subsequent responses. For this reason, each odorant response inhibition value was normalized to the value obtained when the assay was run in the absence of antagonist (sham). The extent of OX1w inhibition varied from 78 ± 5% response remaining for Agam\Orco+Agam\Or65 (activated by eugenol), to 8 ± 1% response remaining for Agam\Orco+Agam\Or28 (activated by acetophenone). While the extent of inhibition varied, which may represent a difference in the allosteric coupling between Orco and each of the various odorant specificity subunits, it is important to note that OX1w was able to inhibit odorant activation of each of the tested ORs. This result suggested that OX1w would be able to interfere with OR mediated behavior, regardless of which odorant specificity subunit and which species is involved (because the highly conserved Orco subunit is a component of all ORs).

### *D*. *melanogaster* larvae display robust attraction to ethyl acetate

**Fig 3:**
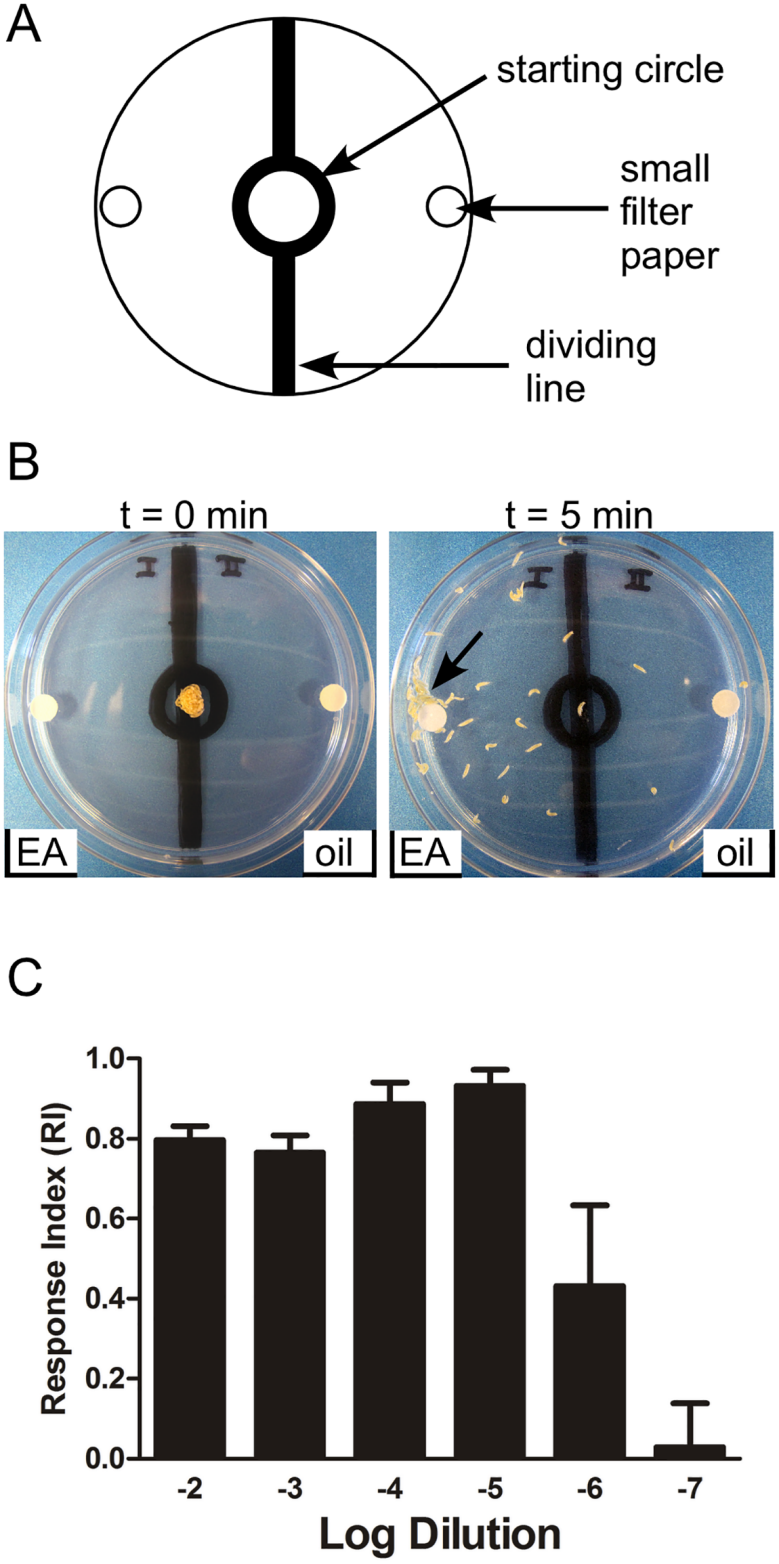
*D*. *melanogaster* larvae are attracted to ethyl acetate (EA). (A) Diagram of larval plate assay. A starting circle was drawn at the center of a 100x15 mm polystyrene Petri dish (VWR) containing 20 ml of 1.1% agarose. A line divides the plate in half, with a small filter disk on each side. A Response Index (RI) is calculated as RI = (S − C)/(S + C), where S = number of larvae on the stimulus (EA) side and C = number of larvae on the control (vehicle) side. RI = 1 would indicate complete attraction, RI = 0 would indicate no preference, and RI = -1 would indicate complete repulsion. (B) Left panel, at the start of an experiment, larvae are in the starting circle flanked on either side by small filter disks. 10 μL of 1:1000 diluted EA was placed on the left filter disk and 10 μL of mineral oil (vehicle) was placed on the right filter disk. Right panel, the same plate, following a 5 min migration period. The majority of larvae have moved towards the filter disk containing EA. A large group of larvae is indicated by the arrow. (C) Larval chemotaxis towards EA is assayed at a series of dilutions. Data are presented as mean ± SEM (n = 4–7).

**Fig 4:**
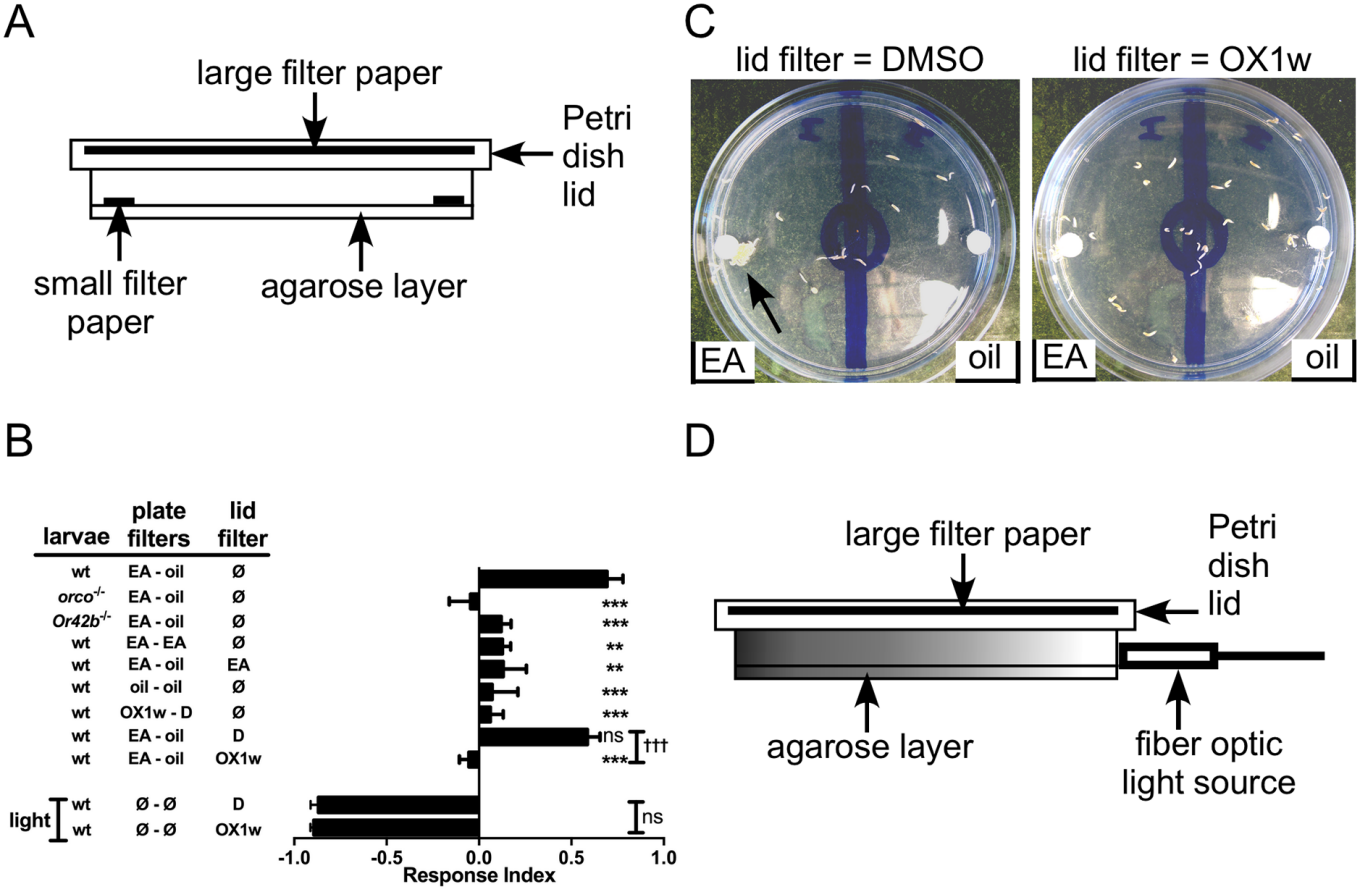
Ethyl acetate attraction is inhibited by an airborne Orco antagonist. (A) Cross-section diagram of the larval plate assay with the addition of a large filter paper on the inner side of the lid. (B) Results of the larval chemotaxis assay. EA, ethyl acetate; oil, mineral oil (vehicle); D, DMSO (vehicle); OX1w, Orco antagonist; Ø, nothing added; light, fiber optic light source. Data are presented as mean ± SEM (n = 4–9). Results were compared by one-way ANOVA and Bonferroni’s multiple comparison test: for comparison to oil vs. oil control (sixth bar from top), **, p<0.01; ***, p<0.001. Light repulsion (bottom 2 bars) with DMSO or OX1w in the lid filter was compared by two-tailed, unpaired t-test. (C) A representative OX1w inhibition experiment. In both panels, larvae were placed in the starting circle, flanked on the left by EA and on the right by mineral oil (vehicle). In the left panel, DMSO (vehicle) was applied to the lid filter paper, while in the right panel, OX1w was applied to the lid filter paper. A large group of larvae is indicated by the arrow. (D) Cross-section diagram of the larval plate assay with addition of a fiber optic light source.

To determine whether compound OX1w could alter olfactory behavior, we turned to a well-established *D*. *melanogaster* larval olfactory chemotaxis assay [53]. A 10 cm plastic culture dish, with the bottom coated with 20 mL of 1.1% agarose, served as the behavioral chamber. Fifty *D*. *melanogaster* larvae (third-instar) were collected and placed in the center of the plate, flanked on either side by small filter discs containing odorant (ethyl acetate) or vehicle (mineral oil). Larvae were allowed to move for 5 min in a dark, quiet chamber before their positions were photographed. Calculation of a Response Index (RI) then allowed assessment of attraction (or repulsion) to the odorant. We chose ethyl acetate (EA) as an attractant because *D*. melanogaster larvae exhibit a strong attraction that is conserved across various strains [53]. A 1:1000 dilution of EA exerted a strong attraction (Fig 3B). To determine an appropriately attractive, but sub-saturating dilution of EA, we tested dilutions ranging from 1:100 to 1:10,000,000 (Fig 3C). Attraction towards EA fell sharply between the 1:100,000 and 1:1,000,000 dilutions, thus we chose a 1:250,000 dilution for subsequent experiments. Larvae were attracted to the 1:250,000 EA dilution with an RI of 0.69 ± 0.09 (Fig 4B), significantly differing from the mineral oil vs. mineral oil control in which the larvae showed no preference (RI = -0.01 ± 0.16).

We used genetically modified larvae to determine whether EA attraction is OR mediated. In contrast to wild type larvae (wt) that were strongly attracted to EA, larvae lacking Orco (*orco* ^-/-^) were not attracted to EA (Fig 4B). Deletion of Dmel\Or42b has been shown to abolish attraction to highly diluted EA [54]. Concordantly, we found that larvae lacking Dmel\Or42b were not attracted to EA. Thus, attraction to a 1:250,000 dilution of EA in our assay is an OR mediated behavior. Placement of EA was alternated between the left and right side of the plate to obviate bias. When EA was placed on both filter discs, larvae distributed equally, showing no preference. Next, we added an additional dimension to the experiment. As demonstrated by Kreher et al. [54], a large filter paper mounted in the inner lid of the dish can serve as a platform with which to test the effect of an additional compound on attraction to the initial compound. To test this arrangement, we added EA to the large filter (685 μL of the 1:100 dilution). Under these conditions, the larvae showed no preference for the 1:250,000 dilution of EA on the small filter disc. This effect, known as masking, was similar to what has been reported [54].

### Attraction to ethyl acetate is abolished by Orco antagonist OX1w

Before testing whether OX1w has an effect on attraction to EA, we first tested whether OX1w alone might evoke a behavioral response. When tested against vehicle (DMSO), we found that OX1w was not attractive or repulsive, with the larvae showing no preference. Thus, under these conditions, OX1w itself does not function as an attractant or repellent.

Next, we assessed the ability of OX1w to act as a behavioral antagonist. We used the lid filter paper configuration shown in Fig 4A. When vehicle alone (685 μL of DMSO) was placed on the lid filter, the larvae remained robustly attracted to EA. In contrast, when 685 μL of 100 mM OX1w was applied to the lid filter, EA attraction was completely abolished (Fig 4B and 4C).

While we have shown that OX1w by itself is not attractive or repulsive to the larvae, it is possible that OX1w might exert a non-OR mediated effect that confuses the larvae or alters sensory perception in some generalized way. Such an effect could confound our assay. To test for this possibility, we used *D*. *melanogaster* larval aversion to light [55]. We used the same assay configuration shown in Fig 4A, with the addition of a light source (Fig 4D). A fiber optic light source was used to minimize thermal effects. First, DMSO (vehicle for OX1w) was placed on the lid filter. Larvae were strongly repelled by the light and moved to the opposite side of the plate, yielding a large negative RI value. The experiment was then repeated with 685 μL of 100 mM OX1w placed on the lid filter. The larvae remained strongly repelled by the light and moved to the opposite side of the plate even in the presence of OX1w. This result demonstrated that OX1w is not exerting a generalized behavioral effect on the larvae. Thus, the Orco antagonist OX1w, applied in an airborne context, can inhibit olfactory behavior in an insect.

Large, non-volatile Orco ligands have been previously shown to be active *in vivo*. When Orco agonist VUAA1 was introduced via a glass recording electrode into capitate peg sensilla on the maxillary palp of *A*. *gambiae*, the two OR expressing OSNs in these sensilla responded with increased spike frequency [38]. When bath applied to mosquito larvae, VUAA1 caused an increase in larval movement that was Orco dependent [56]. Importantly, introduction of Orco antagonist VU0183254 into an *A*. *gambiae* capitate peg sensilla inhibited the OSN response to subsequently applied odorant [51]. Thus, the ability of Orco antagonists to allosterically inhibit odorant activation of ORs that has been demonstrated in heterologous systems [48–51, 57], can also be observed in an *in vivo* context [51]. We have now provided the first demonstration that an Orco antagonist can be applied in an airborne context to alter an OR-mediated insect behavior. These results support the targeting of Orco as a novel approach to the control deleterious insect populations and suggest further development of Orco ligands as a priority.

The apparently ubiquitous presence of Orco in all insect ORs and the high conservation of Orco across insect species makes Orco an appealing target for the development of compounds that can control a wide range of disease vector insects through interference with their olfactory processes. However, widespread and indiscriminant use of such Orco directed compounds would be a cause for concern [58, 59]. Broadly active Orco antagonists would be unsuitable for agricultural use, as both pests and pollinators would be affected. For similar reasons, release of Orco directed compounds over large areas for disease vector control would be problematic. Orco antagonists should be designed and used as short-range deterrents for preventing insect bites as a means to combat insect-borne diseases in humans. Application to skin and clothing would limit the effects to only those insects approaching these sources. Most insects are not attracted to humans and typically do not come in contact with humans, except in self-defense. In this way, improved control of insect-borne diseases might be achieved without generating widespread ecological problems.

## Materials and methods

### Materials

Odorants, Orco ligands, and other chemicals were from Sigma-Aldrich. Dmel\Or35a and Dmel\Orco were generously provided by J. Carlson and L. Vosshall, respectively. Agam\Or15, Agam\Or27, Agam\Or28, Agam\Or31, Agam\Or39, Agam\Or65 in pSP64T-Oligo, and Agam\Orco in pT7TS [18], were generously provided by L. Zwiebel. Cqui\Or21 and Cqui\Orco were obtained as previously described [34, 35] and inserted into pGEMHE [60].

New Orco antagonists tested in this study (with CAS numbers where available) are: OX1t (313499-95-5), N-(2,4-difluorophenyl)-2-thiophenecarboxamide; OX1u, N-(2-chloro-4-fluorophenyl)-2-thiophenecarboxamide; OX1v, N-(3,5-difluorophenyl)-2-thiophenecarboxamide; OX1w (853328-86-6), 3-chloro-N-(4-fluorophenyl)-2-thiophenecarboxamide; OX1x, N-(4-((difluoromethyl)thio)phenyl)-2-thiophenecarboxamide.

### Care and use of *Xenopus laevis* frogs

Mature, female *X*. laevis frogs were used as a source of oocytes for this study. The care and use of *X*. *laevis* frogs was carried out in accordance with the "Guidelines for Egg and Oocyte Harvesting in Xenopus laevis, Revised 07/14/10" from the Animal Research Advisory Committee of the Office of Animal Care and Use at the National Institutes of Health. The protocol was approved by the Institutional Animal Care and Use Committee of the University of Miami (Protocol Numbers: 13–056 and 13–149). Frogs were anesthetized in 0.1% 3-aminobenzoic acid ethyl ester. Sedation was assessed by loss of nasal flare and swallow reflexes. Oocytes were surgically removed and the incision was sutured. Immediately following surgery, a subcutaneous injection of Baytril (0.05 mL of a 2.27% solution) was administered as an antibiotic and one subcutaneous injection of Meloxicam (0.1 mL of a 0.015% solution) was administered to the dorsal lymph sack to serve as an analgesic. Frogs recovered from surgery in a humid environment before being returned to the holding tank. Frogs had a rest period of at least 3 months between surgeries.

### Expression of insect ORs in *Xenopus laevis* oocytes

Follicle cells were removed from the oocytes by a 2 hr treatment with collagenase B (Roche). mMessage mMachine kits (Thermo Fisher Scientific) were used to synthesize capped cRNA for each OR subunit. 25 ng of each OR subunit was injected into stage V-VI oocytes. Oocytes were incubated at 18°C in Barth’s saline (in mM: 88 NaCl, 1 KCl, 2.4 NaHCO3, 0.3 CaNO3, 0.41 CaCl2, 0.82 MgSO4, 15 HEPES pH 7.4, and 0.05 g/L tetracycline, 0.05 g/L ciprofloxacin, 0.1 g/L amikacin) for 2–5 days prior to electrophysiological recording.

### Electrophysiology and data capture

Odorant and Orco ligand induced currents were recorded under two-electrode voltage clamp, using an automated parallel electrophysiology system (OpusExpress 6000A, Molecular Devices). Oocytes were perfused with ND96 (in mM: 96 NaCl, 2 KCl, 1 CaCl2, 1 MgCl2, 5 HEPES, pH 7.4). Odorants and Orco ligands were prepared as 100 mM stock solutions in DMSO and then diluted into ND96 on the day of the experiment. Unless otherwise noted, applications were for 60 sec at a flow rate of 1.0 mL/min, with extensive washing in ND96 at 4.6 mL/min between applications. Micropipettes were filled with 3 M KCl and had resistances of 0.2–2.0 MΩ. The holding potential was -70 mV. Current responses were filtered (4-pole, Bessel, low pass) at 20 Hz (-3 db) and sampled at 100 Hz. Current responses were captured and stored using OpusXpress 1.1 software (Molecular Devices).

### Experimental protocols and data analysis

Antagonist activity at Orco was assessed by exposing oocytes to two 60 sec applications of the Orco agonist OLC12 (2-((4-Ethyl-5- (4-pyridinyl)-4H-1,2,4-triazol-3-yl)sulfanyl)-N-(4-isopropylphenyl)acetamide) with 5 min washes between applications. Oocytes were then exposed to a 90 sec application of antagonist candidate, immediately followed by a 60 sec co-application of antagonist candidate and OLC12. The 90 sec pre-application of antagonist candidate is intended to avoid variation due to differences in antagonist candidate wash on rate [48–50]. The current response in the presence of antagonist candidate was compared to the mean of the preceding two responses to OLC12 alone and presented as a percentage [48].

To measure the ability of Orco antagonist OX1w to inhibit odorant activation, oocytes were exposed to a 30 sec application of odorant followed by a 20 min wash. Oocytes were then exposed to a 90 sec application of OX1w, immediately followed by a 30 sec co-application of OX1w and odorant. The current response in the presence of OX1w was compared to the preceding response to odorant alone and represented as a percentage. Repeated odorant application to ORs expressed in *X*. *laevis* oocytes can cause a substantial decrease in the amplitude of subsequent responses. For this reason, each odorant response inhibition value was normalized to the value obtained when the assay was run in the absence of antagonist (sham).

Initial analysis of electrophysiological data was performed with Clampfit 9.1 software (Molecular Devices). Curve fitting was done using Prism 5 (Graphpad). Concentration-inhibition data were fit to the equation: I =  I_max_/(1+(X/IC_50_)^n^) where I represents the response to activator in the presence of a given concentration of inhibitor, X; I_max_ is the maximal response to activator in the absence of inhibitor; IC_50_ is the concentration of inhibitor that reduces the response to activator by 50%; n is the apparent Hill coefficient.

### *Drosophila* larvae care and chemotaxis assay

Canton-S (CS) flies were used as wild type (wt). Mutant flies (w*; *TI{TI}Orco*^*2*^) and (*y*^*1*^ *w*^*67c23*^; *P{EPgy2}Or42b*^*EY14886*^) were obtained from the Bloomington stock center. Larvae were kept in a controlled environment with a temperature of 20–26°C and a relative humidity of 60–80%. They were housed in 95x28 mm polypropylene vials (VWR) topped with cotton and contained 10 mL of food media. Food media consisted of agar, molasses, corn meal, dried yeast, propionic acid, and p-hydroxybenzoic acid methyl ester.

To harvest larvae for experiments, food media containing larvae was scooped out of the housing vial and placed in a Petri dish. Distilled water was then vigorously applied onto the clump to separate the larvae from the media and the plate was shaken in a circular motion to further promote the separation. Forceps were used to pick up each individual larva and place it in a small droplet of water located in a fresh Petri dish. Following the collection of 50 larvae, the flat edge of the scoop was used to push the larvae to one side of the water droplet and a Kimwipe was used on the opposite side to soak up as much water as possible. A “ball” of dried larvae was then placed in the center of the experimental dish.

The experimental dish was a 100x15 mm polystyrene Petri dish (VWR) containing 20 mL of 1.1% agarose. The larvae were placed inside a “starting circle” that was drawn on the outside bottom of the plate. A line was also drawn that divided the plate in half. Two small filter paper disks (d = 6 mm) were place on the agarose surface, one on each side of the plate (Fig 3A). These small filter paper disks were punched from Whatman Cellulose Filter Papers (Cat. No. 1030 023). Onto each filter paper disk was placed 10 μL of solution. Solution contained either vehicle of dilution (mineral oil or DMSO) or an odorant or antagonist compound (diluted in vehicle).

For odor masking and antagonist assays, we used the method described by Kreher et al. [54]. A large (d = 9 cm) filter paper (Whatman Filter Paper Cat. No. D00161-S) was adhered with double-sided tape to the inside of the Petri dish lid. Kreher et al. [54] reported that 750 μL could be placed on the lid filter without dripping onto the plate. In pilot experiments, we tested volumes from 500 μL to 1000 μL in 50 μL increments and found that 850 μL could be placed on the lid filter without dripping. To be cautious, we decided to use a volume of 700 μL in these experiments. However, at the time of experimentation, the amount of OX1w compound available required us to further reduce the volume to 685 μL per experiment. Antagonist candidate (OX1w) was screened at a 100 mM concentration. Once larvae were placed in the center of the dish, the solutions were applied to the filter papers and the lid was placed on top of the dish. A box was then placed over the entire experiment to block out light. Additionally, the box was insulated with Styrofoam and the entire experiment took place on top of Styrofoam to insulate from noise and vibrations. Following a 5 minute period during which the larvae were allowed to migrate, the box was removed and a photograph was taken to document the location of the larvae on the plate. Each photograph was coded and then counted blindly by a different individual.

Light aversion experiments were done in a manner similar to the olfactory attraction assay. These experiments did not include the small filter papers. Larvae were placed in the center of the experimental dish, solution was applied to the large lid filter paper, and the lid was closed. The entire experiment was covered with a box, and a fiber optic light was then turned on. The light was placed such that it shown across the plane of the agarose and hit the larvae at a 0° incidence angle. The light source (Bausch and Lomb Fiber Lite) was set at 2/3 intensity. We also explored the possibility of using compounds that activate the ionotropic (IR) class of insect olfactory receptors in the chemotaxis assay. These receptors are structurally similar to ionotropic glutamate receptors [61] and thus should not be sensitive to Orco antagonist compounds. IRs have been reported to respond to a variety of compounds, such as phenylacetaldehyde, phenethylamine, ammonia and acetic acid [61, 62]. We tested 27 reported or potential IR ligands in the chemotaxis assay at 1:100 dilutions. However, none of these compounds displayed attraction or repulsion (S5 Table).

A Response Index (RI) was calculated as RI = (S − C)/(S + C), where S is the number of larvae on the stimulus side and C is the number of larvae on the control side. Larvae not leaving the starting circle or touching the dividing line were excluded from the analysis. Statistical significance was assessed using a one-way analysis of the variance followed by Bonferroni’s post-test, or a two-tailed unpaired t-test, as appropriate.

## Supporting information

S1 Fig OX1w inhibition of homomeric Orco channels. Oocytes expressing Dmel\Orco, Cqui\Orco or Agam\Orco were challenged with two 60 sec applications of the Orco agonist OLC12 (30 μM) with a 5 min wash between applications. Oocytes were then exposed to a 90 sec application of 100 μM OX1w, immediately followed by a 60 sec co-application of OX1w and OLC12. The current response in the presence of OX1w was compared to the mean of the preceding two responses to OLC12 alone and presented as a percentage (mean ± SEM, n = 5–8). The underlying data for this figure may be found in S6 Table.
(PDF)

S1 Table Data underlying Fig 1. (XLSX)

S2 Table Data underlying Fig 2. (XLSX)

S3 Table Data underlying Fig 3. (XLSX)

S4 Table Data underlying Table 1. (XLSX)

S5 Table Data for screening of potential IR active compounds. (XLSX)

S6 Table Data underlying S1 Fig. (XLSX)

## References

[1] Gibson G, Torr SJ. Visual and olfactory responses of haematophagous Diptera to host stimuli. Med Vet Entomol. 1999;13: 2–23. 10194745 10.1046/j.1365-2915.1999.00163.x

[2] Carey AF, Carlson JR. Insect olfaction from model systems to disease control. Proc Natl Acad Sci USA. 2011;108: 12987–12995. 10.1073/pnas.1103472108 21746926 PMC3156210

[3] Pickett JA, Birkett MA, Dewhirst SY, Logan JG, Omolo MO, Torto B, et al Chemical ecology of animal and human pathogen vectors in a changing global climate. J Chem Ecol. 2010;36: 113–121. 10.1007/s10886-010-9739-9 20119869

[4] Sato K, Pellegrino M, Nakagawa T, Vosshall LB, Touhara K. Insect olfactory receptors are heteromeric ligand-gated ion channels. Nature. 2008;452: 1002–1006. 10.1038/nature06850 18408712

[5] Wicher D, Schafer R, Bauernfeind R, Stensmyr MC, Heller R, Heinemann SH, et al Drosophila odorant receptors are both ligand-gated and cyclic-nucleotide-activated cation channels. Nature. 2008;452: 1007–1011. 10.1038/nature06861 18408711

[6] Nakagawa T, Vosshall LB. Controversy and consensus: noncanonical signaling mechanisms in the insect olfactory system. Curr Opin Neurobiol. 2009;19: 284–292. 10.1016/j.conb.2009.07.015 19660933 PMC2752668

[7] Benton R, Sachse S, Michnick SW, Vosshall LB. Atypical membrane topology and heteromeric function of Drosophila odorant receptors in vivo. PLoS Biol. 2006;4: e20 10.1371/journal.pbio.0040020 16402857 PMC1334387

[8] Vosshall LB, Stocker RF. Molecular architecture of smell and taste in Drosophila. Annu Rev Neurosci. 2007;30: 505–533. 10.1146/annurev.neuro.30.051606.094306 17506643

[9] Vosshall LB, Hansson BS. A unified nomenclature system for the insect olfactory coreceptor. Chem Senses. 2011;36:497–498. 10.1093/chemse/bjr022 21441366

[10] Nakagawa T, Pellegrino M, Sato K, Vosshall LB, Touhara K. Amino acid residues contributing to function of the heteromeric insect olfactory receptor complex. PloS One. 2012;7: e32372 10.1371/journal.pone.0032372 22403649 PMC3293798

[11] Nichols AS, Chen S, Luetje CW. Subunit contributions to insect olfactory receptor function: channel block and odorant recognition. Chem Senses. 2011;36: 781–790. 10.1093/chemse/bjr053 21677030 PMC3195787

[12] Pask GM, Jones PL, Rutzler M, Rinker DC, Zwiebel LJ. Heteromeric anopheline odorant receptors exhibit distinct channel properties. PloS One. 2011;6: e28774 10.1371/journal.pone.0028774 22174894 PMC3235152

[13] Carey AF, Wang G, Su CY, Zwiebel LJ, Carlson JR. Odorant reception in the malaria mosquito Anopheles gambiae. Nature. 2010;464: 66–71. 10.1038/nature08834 20130575 PMC2833235

[14] Hallem EA, Carlson JR. Coding of odors by a receptor repertoire. Cell. 2006;125: 143–160. 10.1016/j.cell.2006.01.050 16615896

[15] Hallem EA, Ho MG, Carlson JR. The molecular basis of odor coding in the Drosophila antenna. Cell. 2004;117: 965–979. 10.1016/j.cell.2004.05.012 15210116

[16] Nichols AS, Luetje CW. Transmembrane segment 3 of Drosophila melanogaster odorant receptor subunit 85b contributes to ligand-receptor interactions. J Biol Chem. 2010;285: 11854–11862. 10.1074/jbc.M109.058321 20147286 PMC2852922

[17] Pellegrino M, Steinbach N, Stensmyr MC, Hansson BS, Vosshall LB. A natural polymorphism alters odour and DEET sensitivity in an insect odorant receptor. Nature. 2011;478: 511–514. 10.1038/nature10438 21937991 PMC3203342

[18] Wang G, Carey AF, Carlson JR, Zwiebel LJ. Molecular basis of odor coding in the malaria vector mosquito Anopheles gambiae. Proc Natl Acad Sci USA. 2010;107: 4418–4423. 10.1073/pnas.0913392107 20160092 PMC2840125

[19] Leal WS, Choo YM, Xu P, da Silva CS, Ueira-Vieira C. Differential expression of olfactory genes in the southern house mosquito and insights into unique odorant receptor gene isoforms. Proc Natl Acad Sci USA. 2013;110: 18704–18709. 10.1073/pnas.1316059110 24167245 PMC3831946

[20] Krieger J, Klink O, Mohl C, Raming K, Breer H. A candidate olfactory receptor subtype highly conserved across different insect orders. J Comp Physiol A Neuroethol Sens Neural Behav Physiol. 2003;189: 519–526. 10.1007/s00359-003-0427-x 12827420

[21] Larsson MC, Domingos AI, Jones WD, Chiappe ME, Amrein H, Vosshall LB. Or83b encodes a broadly expressed odorant receptor essential for Drosophila olfaction. Neuron. 2004;43: 703–714. 10.1016/j.neuron.2004.08.019 15339651

[22] Nakagawa T, Sakurai T, Nishioka T, Touhara K. Insect sex-pheromone signals mediated by specific combinations of olfactory receptors. Science. 2005;307: 1638–1642. 10.1126/science.1106267 15692016

[23] Neuhaus EM, Gisselmann G, Zhang W, Dooley R, Stortkuhl K, Hatt H. Odorant receptor heterodimerization in the olfactory system of Drosophila melanogaster. Nat Neurosci. 2005;8: 15–17. 10.1038/nn1371 15592462

[24] Pitts RJ, Fox AN, Zwiebel LJ. A highly conserved candidate chemoreceptor expressed in both olfactory and gustatory tissues in the malaria vector Anopheles gambiae. Proc Natl Acad Sci USA. 2004;101: 5058–5063. 10.1073/pnas.0308146101 15037749 PMC387373

[25] Wanner KW, Nichols AS, Walden KK, Brockmann A, Luetje CW, Robertson HM. A honey bee odorant receptor for the queen substance 9-oxo-2-decenoic acid. Proc Natl Acad Sci USA. 2007;104: 14383–14388. 10.1073/pnas.0705459104 17761794 PMC1964862

[26] Dweck HK, Ebrahim SA, Farhan A, Hansson BS, Stensmyr MC. Olfactory proxy detection of dietary antioxidants in Drosophila. Curr Biol. 2015;25: 455–466. 10.1016/j.cub.2014.11.062 25619769

[27] Stensmyr MC, Dweck HK, Farhan A, Ibba I, Strutz A, Mukunda L, et al A conserved dedicated olfactory circuit for detecting harmful microbes in Drosophila. Cell. 2012;151: 1345–1357. 10.1016/j.cell.2012.09.046 23217715

[28] DeGennaro M. The mysterious multi-modal repellency of DEET. Fly (Austin). 2015;9: 45–51. 26252744 10.1080/19336934.2015.1079360 PMC4594586

[29] Leal WS. The enigmatic reception of DEET—the gold standard of insect repellents. Curr Opin Insect Sci. 2014;6: 93–98. 10.1016/j.cois.2014.10.007 25530943 PMC4269249

[30] Stanczyk NM, Brookfield JF, Field LM, Logan JG. Aedes aegypti mosquitoes exhibit decreased repellency by DEET following previous exposure. PloS One. 2013;8: e54438 10.1371/journal.pone.0054438 23437043 PMC3577799

[31] Sparks JT, Dickens JC. Bitter-sensitive gustatory receptor neuron responds to chemically diverse insect repellents in the common malaria mosquito Anopheles quadrimaculatus. Die Naturwissenschaften. 2016;103: 39 10.1007/s00114-016-1367-y 27108454

[32] Lee Y, Kim SH, Montell C. Avoiding DEET through Insect Gustatory Receptors. Neuron. 2010;67: 555–561. 10.1016/j.neuron.2010.07.006 20797533 PMC2929391

[33] DeGennaro M, McBride CS, Seeholzer L, Nakagawa T, Dennis EJ, Goldman C, et al orco mutant mosquitoes lose strong preference for humans and are not repelled by volatile DEET. Nature. 2013;498: 487–491. 10.1038/nature12206 23719379 PMC3696029

[34] Hughes DT, Pelletier J, Luetje CW, Leal WS. Odorant receptor from the southern house mosquito narrowly tuned to the oviposition attractant skatole. J Chem Ecol. 2010;36: 797–800. 10.1007/s10886-010-9828-9 20623327 PMC2908433

[35] Pelletier J, Hughes DT, Luetje CW, Leal WS. An odorant receptor from the southern house mosquito Culex pipiens quinquefasciatus sensitive to oviposition attractants. PloS One. 2010;5: e10090 10.1371/journal.pone.0010090 20386699 PMC2851645

[36] Xia Y, Wang G, Buscariollo D, Pitts RJ, Wenger H, Zwiebel LJ. The molecular and cellular basis of olfactory-driven behavior in Anopheles gambiae larvae. Proc Natl Acad Sci USA. 2008;105: 6433–6438. 10.1073/pnas.0801007105 18427108 PMC2359781

[37] Xu P, Choo YM, De La Rosa A, Leal WS. Mosquito odorant receptor for DEET and methyl jasmonate. Proc Natl Acad Sci USA. 2014;111: 16592–16597. 10.1073/pnas.1417244111 25349401 PMC4246313

[38] Jones PL, Pask GM, Rinker DC, Zwiebel LJ. Functional agonism of insect odorant receptor ion channels. Proc Natl Acad Sci USA. 2011;108: 8821–8825. 10.1073/pnas.1102425108 21555561 PMC3102409

[39] Rinker DC, Jones PL, Pitts RJ, Rutzler M, Camp G, Sun LJ, et al Novel high-throughput screens of Anopheles gambiae odorant receptors reveal candidate behavior-modifying chemicals for mosquitoes. Physiol Entomol. 2012;37: 33–41. 10.1111/j.1365-3032.2011.00821.x PMC7123412 32255891

[40] Ramdya P, Benton R. Evolving olfactory systems on the fly. Trends Genet. 2010;26(7):307–16. 10.1016/j.tig.2010.04.004 20537755

[41] Siju KP, Hill SR, Hansson BS, Ignell R. Influence of blood meal on the responsiveness of olfactory receptor neurons in antennal sensilla trichodea of the yellow fever mosquito, Aedes aegypti. J Insect Physiol. 2010;56: 659–665. 10.1016/j.jinsphys.2010.02.002 20153749

[42] Smallegange RC, van Gemert GJ, van de Vegte-Bolmer M, Gezan S, Takken W, Sauerwein RW, et al Malaria infected mosquitoes express enhanced attraction to human odor. PloS One. 2013;8: e63602 10.1371/journal.pone.0063602 23691073 PMC3655188

[43] Takken W, van Loon JJ, Adam W. Inhibition of host-seeking response and olfactory responsiveness in Anopheles gambiae following blood feeding. J Insect Physiol. 2001;47: 303–310. 11119776 10.1016/s0022-1910(00)00107-4

[44] Fox AN, Pitts RJ, Robertson HM, Carlson JR, Zwiebel LJ. Candidate odorant receptors from the malaria vector mosquito Anopheles gambiae and evidence of down-regulation in response to blood feeding. Proc Natl Acad Sci U S A. 2001;98: 14693–14697. 10.1073/pnas.261432998 11724964 PMC64743

[45] Jones WD, Nguyen TA, Kloss B, Lee KJ, Vosshall LB. Functional conservation of an insect odorant receptor gene across 250 million years of evolution. Curr Biol. 2005;15: R119–121. 10.1016/j.cub.2005.02.007 15723778

[46] Li Y, Zhang J, Chen D, Yang P, Jiang F, Wang X, et al CRISPR/Cas9 in locusts: Successful establishment of an olfactory deficiency line by targeting the mutagenesis of an odorant receptor co-receptor (Orco). Insect Biochem Mol Biol. 2016;79: 27–35. 10.1016/j.ibmb.2016.10.003 27744049

[47] Franco TA, Oliveira DS, Moreira MF, Leal WS, Melo AC. Silencing the odorant receptor co-receptor RproOrco affects the physiology and behavior of the Chagas disease vector Rhodnius prolixus. Insect Biochem Mol Biol. 2016;69: 82–90. 10.1016/j.ibmb.2015.02.012 25747010

[48] Chen S, Luetje CW. Identification of new agonists and antagonists of the insect odorant receptor co-receptor subunit. PloS One. 2012;7: e36784 10.1371/journal.pone.0036784 22590607 PMC3348135

[49] Chen S, Luetje CW. Phenylthiophenecarboxamide antagonists of the olfactory receptor co-receptor subunit from a mosquito. PloS One. 2013;8: e84575 10.1371/journal.pone.0084575 24358366 PMC3866151

[50] Chen S, Luetje CW. Trace amines inhibit insect odorant receptor function through antagonism of the co-receptor subunit. F1000Res. 2014;3: 84 10.12688/f1000research.3825.1 25075297 PMC4097363

[51] Jones PL, Pask GM, Romaine IM, Taylor RW, Reid PR, Waterson AG, et al Allosteric antagonism of insect odorant receptor ion channels. PloS One. 2012;7: e30304 10.1371/journal.pone.0030304 22272331 PMC3260273

[52] Tsitoura P, Koussis K, Iatrou K. Inhibition of Anopheles gambiae odorant receptor function by mosquito repellents. J Biol Chem. 2015;290: 7961–7972. 10.1074/jbc.M114.632299 25657000 PMC4367294

[53] Monte P, Woodard C, Ayer R, Lilly M, Sun H, Carlson J. Characterization of the larval olfactory response in Drosophila and its genetic basis. Behav Gen. 1989;19: 267–283. 10.1007/BF01065910 2497723

[54] Kreher SA, Mathew D, Kim J, Carlson JR. Translation of sensory input into behavioral output via an olfactory system. Neuron. 2008;59: 110–124. 10.1016/j.neuron.2008.06.010 18614033 PMC2496968

[55] Sawin-McCormack EP, Sokolowski MB, Campos AR. Characterization and genetic analysis of Drosophila melanogaster photobehavior during larval development. J Neurogenet. 1995;10: 119–235. 8592272 10.3109/01677069509083459

[56] Taylor RW, Romaine IM, Liu C, Murthi P, Jones PL, Waterson AG, et al Structure-activity relationship of a broad-spectrum insect odorant receptor agonist. ACS Chem Biol. 2012;7: 1647–1652. 10.1021/cb300331z 22924767

[57] Tsitoura P, Andronopoulou E, Tsikou D, Agalou A, Papakonstantinou MP, Kotzia GA, et al Expression and membrane topology of Anopheles gambiae odorant receptors in lepidopteran insect cells. PloS One. 2010;5: e15428 10.1371/journal.pone.0015428 21082026 PMC2972716

[58] Andersson MN, Newcomb RD. Pest control compounds targeting insect chemoreceptors: Another silent spring? Front Ecol Evol. 2017;5: 5.

[59] Sutherland WJ, Clout M, Depledge M, Dicks LV, Dinsdale J, Entwistle AC, et al A horizon scan of global conservation issues for 2015. Trends Ecol Evol. 2015;30: 17–24. 10.1016/j.tree.2014.11.002 25433442

[60] Liman ER, Tytgat J, Hess P. Subunit stoichiometry of a mammalian K+ channel determined by construction of multimeric cDNAs. Neuron. 1992;9: 861–871. 1419000 10.1016/0896-6273(92)90239-a

[61] Benton R, Vannice KS, Gomez-Diaz C, Vosshall LB. Variant ionotropic glutamate receptors as chemosensory receptors in Drosophila. Cell. 2009;136: 149–162. 10.1016/j.cell.2008.12.001 19135896 PMC2709536

[62] Abuin L, Bargeton B, Ulbrich MH, Isacoff EY, Kellenberger S, Benton R. Functional architecture of olfactory ionotropic glutamate receptors. Neuron. 2011;69: 44–60. 10.1016/j.neuron.2010.11.042 21220098 PMC3050028

