# Correction: Inhibition of insect olfactory behavior by an airborne antagonist of the insect odorant receptor co-receptor subunit

**DOI:** 10.1371/journal.pone.0183009

**Published:** 2017-08-03

**Authors:** Devin Kepchia, Scott Moliver, Kunal Chohan, Cameron Phillips, Charles W. Luetje

The image for Fig 4B is incorrect. Please see the correct Fig 4 here.

**Fig 4:**
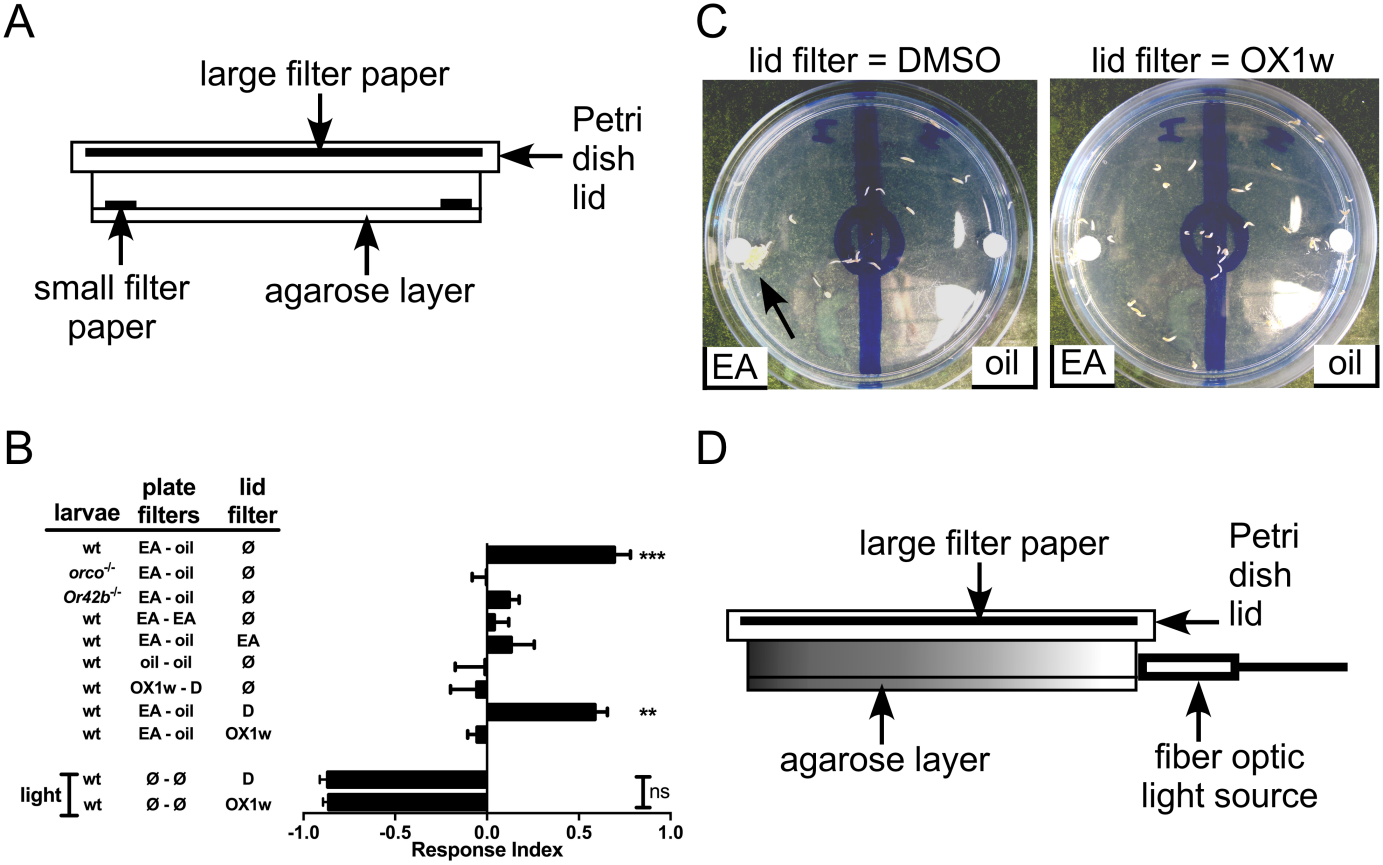
Ethyl acetate attraction is inhibited by an airborne Orco antagonist. (A) Cross-section diagram of the larval plate assay with the addition of a large filter paper on the inner side of the lid. (B) Results of the larval chemotaxis assay. EA, ethyl acetate; oil, mineral oil (vehicle); D, DMSO (vehicle); OX1w, Orco antagonist; Ø, nothing added; light, fiber optic light source. Data are presented as mean ± SEM (n = 4–9). Results were compared by one-way ANOVA and Bonferroni’s multiple comparison test: for comparison to oil vs. oil control (sixth bar from top), **, p<0.01; ***, p<0.001. Light repulsion (bottom 2 bars) with DMSO or OX1w in the lid filter was compared by two-tailed, unpaired t-test. (C) A representative OX1w inhibition experiment. In both panels, larvae were placed in the starting circle, flanked on the left by EA and on the right by mineral oil (vehicle). In the left panel, DMSO (vehicle) was applied to the lid filter paper, while in the right panel, OX1w was applied to the lid filter paper. A large group of larvae is indicated by the arrow. (D) Cross-section diagram of the larval plate assay with addition of a fiber optic light source.

## References

[1] Kepchia D, Moliver S, Chohan K, Phillips C, Luetje CW (2017) Inhibition of insect olfactory behavior by an airborne antagonist of the insect odorant receptor co-receptor subunit. PLoS ONE 12(5): e0177454 https://doi.org/10.1371/journal.pone.0177454 28562598 10.1371/journal.pone.0177454 PMC5451006

