# Supplementary material for: Inhibition of insect olfactory behavior by an airborne antagonist of the insect odorant receptor co-receptor subunit
Source: PLoS One. 2017 May 31;12(5):e0177454. doi: 10.1371/journal.pone.0177454 (PMC5451006; doi:10.1371/journal.pone.0177454)

S1 Fig. Supplementary Figure: OX1w inhibition of homomeric Orco channels.

Oocytes expressing Dmel\Orco, Cqui\Orco or Agam\Orco were challenged with two 60 sec applications of the Orco agonist OLC12 (30 μM) with a 5 min wash between applications. Oocytes were then exposed to a 90 sec application of 100 μM OX1w, immediately followed by a 60 sec co-application of OX1w and OLC12. The current response in the presence of OX1w was compared to the mean of the preceding two responses to OLC12 alone and presented as a percentage (mean ± SEM, n = 5 - 8). The underlying data for this figure may be found in S6 Table.

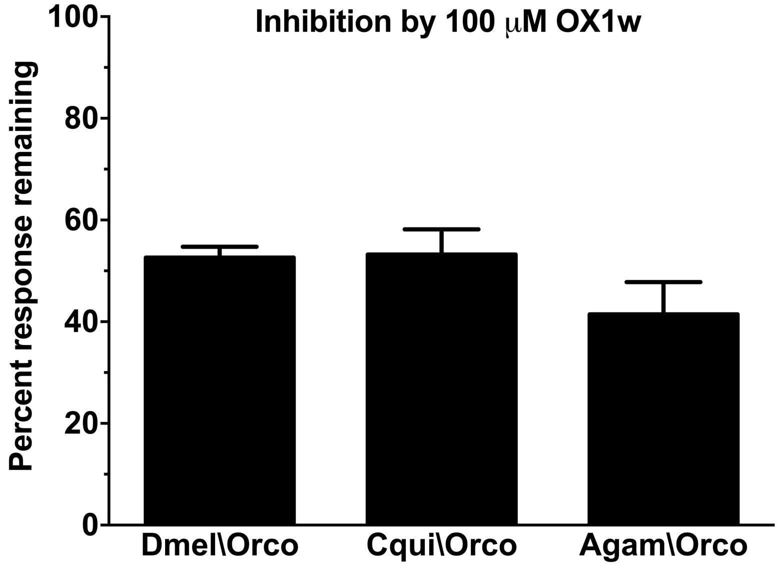

Supplement: S1 Fig — Oocytes expressing Dmel\Orco, Cqui\Orco or Agam\Orco were challenged with two 60 sec applications of the Orco agonist OLC12 (30 μM) with a 5 min wash between applications. Oocytes were then exposed to a 90 sec application of 100 μM OX1w, immediately followed by a 60 sec co-application of OX1w and OLC12. The current response in the presence of OX1w was compared to the mean of the preceding two responses to OLC12 alone and presented as a percentage (mean ± SEM, n = 5–8). The underlying data for this figure may be found in S6 Table. (PDF) [file pone.0177454.s001.pdf]
